# Welcome from the new *Discover Oncology* journal: Editorial - 28th December, 2020

**DOI:** 10.1007/s12672-021-00395-9

**Published:** 2021-01-26

**Authors:** Eleonora Candi

**Affiliations:** University of Rome “Tor Vergata”, Rome, Italy

*Discover Oncology* is a new open access journal published by Springer Nature, embracing all aspects of oncology from basic science to clinical trials. The journal is a continuation and expansion of the journal *Hormones and Cancer*, also from Springer Nature. By re-launching *Discover Oncology* we aim to expand its scope and represent all areas of oncology research by publishing both original work and timely reviews.

In recent years there has been a consistent growth in research in the field of oncology and cancer research, and so *Discover Oncology* aims to provide a unified forum for discussion of this topic. The journal spans from basic and translational science, through preclinical, clinical, and epidemiology, and welcomes content that interfaces at all levels of cancer research. Areas covered include, but are not limited to, cancer causation and the underlying biology of disease, the molecular interactions between genes, hormones, the immune system and the metabolism, screening and prevention, diagnosis and treatment, prognostication, novel therapeutic agents and clinical trials. All cancer types, including benign tumors, will be considered for publication.

The journal has an inclusive scope based on quality, reliability, and rigour, delivered via a transparent and quick peer review process. One of the aims of *Discover Oncology* is to make publishing more efficient, constructive, and fair. We will try to give scientists the opportunity to publish valuable papers with only one set of referee reports and no more than one round of revision, in order to restrict revisions to the essentials. We aim to prevent repetitive, lengthy rounds of review, avoiding referee exhaustion, author frustration, and slow dissemination of research results.

*Discover Oncology’*s editorial process is highly efficient and provides authors with the best editorial experience: dedicated scientific editors and research-active academic experts drive the process with advice from a committed, young academic board. The collaborative editorial process ensures timely, balanced decisions across all subject categories.

As Editor-in-Chief, I am honored to serve the scientific community by leading this new journal. I believe that facilitating the communication of discoveries has an important role for society and the scientific community. *Discover Oncology* will give space not only to established topics in the field, but also to emerging, preliminary studies and discoveries. *Discover Oncology* will also be supportive of early-career researchers, allowing them to publish their studies. Regardless of my personal perspective and scientific background as a molecular biologist, the focus of the journal will be broad. Personally, I will exert all efforts to keep the journal updated and adapted in its editorial process and scope and to meet community expectations so it can become a cutting edge forum for all the different oncology fields. I am eagerly looking forward to sharing our first articles with you. I sincerely welcome you as a reader and author!

Eleonora Candi, PhD.

Editor-in-chief.

